# Fetal Development in Anatomical Preparations of Ruysch and the Meckels in Comparison

**DOI:** 10.3390/ijerph192214896

**Published:** 2022-11-12

**Authors:** Oxana Kosenko, Claudia Steinicke, Heike Kielstein, Florian Steger

**Affiliations:** 1 Institute of the History, Philosophy and Ethics of Medicine at Ulm University, Parkstraße 11, 89073 Ulm, Germany; 2 Institute of Anatomy and Cell Biology, Martin Luther University Halle-Wittenberg, Große Steinstraße 52, 06108 Halle, Germany

**Keywords:** anatomical collection, anatomical specimen, history of anatomy, fetal development, teratology

## Abstract

Anatomical collections have been used for centuries for research and teaching purposes. By the example of selected preparations of fetal development from the Ruysch collection (17th–18th centuries) and the Meckel collections (18th–19th century), this article aims to trace the changing purposes, specifics and methods of creating specimens as well as the development of anatomy during that period. The selected specimens are compared and analyzed implementing the historical-critical method. Regarding the appearance of the preparations, we see a transition from the visually aesthetic specimens (Ruysch) to exact ones (Meckel collections). If Ruysch’s preparations were compared in his time to works of art, specimens created by three anatomists of the Meckel dynasty were primarily created for teaching and research purposes. However, Ruysch’s preparations tracing fetal circulation were scientific discoveries of the time. As for preparations of fetal development from the Meckel collections, we see both specimens of physiological processes already known at that time and experimental ones. Regarding teratology, Ruysch and Meckel the Younger tried to explain malformations, but their anatomical preparations could hardly give answers to the cause of deviations from the norm. The differences between the collections can be explained by the different stages of development of anatomy of the time and by the research interests of the anatomists themselves.

## 1. Introduction

Anatomical collections of medical educational institutions are an indispensable part of research and visual aids for students. The early anatomical collections were also a testament of the skill of the anatomist himself, and even perceived by contemporaries as pieces of art. While some of these collections are stored in museums and belong to the cultural heritage, others are still being used for research and teaching purposes. The first specialized collections of anatomical specimens emerged in the late 16th century and early 17th century, the largest of which were at the Universities in Padua and Leiden but consisted mostly of skeletons [1]. With the paradigm shift in anatomy at the turn of the 17th and 18th centuries and the transition from speculative views to experimental science, there was also a change in anatomical collections. Along with the development of injection techniques and mixtures, anatomists began to create dry and wet preparations, which could show functional processes in the organs.

World famous at the time was the anatomical collection of the Dutch physician Frederick Ruysch (1638–1731), who has been called “the apostle of the injection method” [2]. He became widely known even beyond the Netherlands for his anatomical collection, which contemporaries regarded as “the Eighth Wonder of the World” [3]. Ruysch was appointed professor in anatomy in 1666 and botany in 1685 at the Athenaeum Illustre, the predecessor of the University of Amsterdam, and remained in those positions until his death in 1731. In addition, in 1672–1712 he was the City Obstetrician of Amsterdam obliged to teach midwives anatomy [4]. That post gave him official access to the bodies of stillborn and aborted infants, which contributed to the growth of his anatomical collection. Although the collection was private, he allowed any interested person to see it [5]. It contained over 2,000 embalmed or dried anatomical, pathological, zoological or botanical specimens. The anatomical part of the collection included dry preparations (skeletons, skulls and dried organs) as well as wet preparations. The Russian Tsar Peter the Great (1672–1725) bought the Ruysch collection in 1717, which was shipped to St. Petersburg and became the core of Russia’s first museum, the Kunstkamera, now Peter the Great Museum of Anthropology and Ethnography. The Ruysch collection was to serve the purpose of popularizing reliable, obvious knowledge and the establishment of science and enlightenment in Russia.

Anatomical collections began to spread widely in the second half of the 18th century and at the beginning of the 19th century in Europe and were created for the purpose of teaching and research. The Meckel collections in Halle (Saale) are the most extensive of their kind in Germany and one of the biggest in Europe, with over 8,000 exhibits and are divided into a human-anatomical and a comparative-anatomical collection area [6,7]. The collections include preparations made by three anatomists of the Meckel dynasty. At his time, Johann Friedrich Meckel the Elder (1724–1774) was one of the leading anatomists in Germany. His contemporaries called him the “master of dissection” [6]. In 1750, he became a professor of anatomy at the Collegium medico-chirurgicum in Berlin, later also a professor of botany and obstetrics. Meckel resigned from his posts at the Collegium medico-chirurgicum in 1773, when he was diagnosed with tuberculosis [8]. Following in the long tradition of his colleagues, Meckel also built his own collection of anatomical preparations, which should serve as aids for teaching and research. The exact volume of the collection at that time is unknown, but historians estimate it to be 200–300 anatomical and pathological preparations [6]. It included dry as well wet preparations of organs or body parts, skeletons and skulls. Meckel passed on his anatomical knowledge and skills to his eldest son Philipp Friedrich Theodor (1755–1803) at a very early age. Philipp Meckel was appointed professor of anatomy, surgery and obstetrics at the University of Halle in 1777. He was considered a passionate anatomist who was constantly striving to expand the collection, primarily with specimens of pathological anatomy. His passion went so far that he dissected two of his own three prematurely born children. Moreover, before his death, he had ordered to be dissected, and his assembled skeleton was placed in a special cabinet. His skeleton is still kept in the Meckel collections. Philipp Meckel’s work as an obstetrician deserves special mention. His stellar reputation in the field of childbirth was the reason he was invited to St. Petersburg in 1798 to deliver a baby of Empress Consort of Russia Maria Federovna (1759–1828) [6]. By the time he died in 1803, his collection included 3400 anatomical specimens. He bequeathed it to his youngest son Johann Friedrich Meckel the Younger (1781–1833), who also followed in his grandfather’s and father’s footsteps of being a famous anatomist. In 1808, he was appointed as a full professor of anatomy, surgery and obstetrics at the University of Halle. He resigned from the fields of surgery and obstetrics in 1810 and concentrated his professional activities only on anatomy, physiology and pathological anatomy [6]. He significantly expanded the collection inherited from his father. By 1830, it comprised about 12,000 specimens, and according to his own information, even 16,000. He was not interested in a public viewing and use of his collection but prevented the University of Halle’s plan to build its own collection independently of Meckel. In 1830, he was forced to agree to grant access to the collection to all professors, docents and students of the University. After Meckel’s death, his wife sold the anatomical collection to the University of Halle. Since optimal accommodation of the collection was not provided, it suffered heavy losses. Many preparations broke or became moldy due to storage in heaps or were eaten by moths and skin beetles. In addition, a significant number of specimens were later transferred to the University’s Institute of Pathology [6]. Nowadays, the Meckel collections, which contain about 8000 preparations, are housed in the Institute of Anatomy and Cell Biology at Martin Luther University Halle-Wittenberg and have been Germany’s nationally valuable cultural property since June 2015.

Despite the work of educational institutions and museums in describing their anatomical collections and the history of their creation [1,6,9,10,11], there are only some special generalizing or comparative research papers on the history of anatomical collections in the 17th and 18th centuries [12,13,14,15]. Medical historians have already pointed out that anatomical collections are “fluid”, as the appearance of the specimens and their purpose constantly change according to the cultural background and research interests of their creators [15]. Thus, in this article, we want to compare the Ruysch and the Meckel collections in order to trace how the views of anatomists changed from the end of the 17th to the beginning of the 19th century and what impact these collections had on the further development of science. Through the example not only of the history of these collections, but also of their selected preparations of fetal development, our paper aims to answer the following questions: What was the purpose of the collection of anatomical specimens? What did the anatomists want to show in the preparations, and what principles did they use to construct an anatomical collection? What differences do we observe between both collections, and what do they tell us about the development of anatomy in that period?

## 2. Materials and Methods

Research works of Ruysch and anatomists from the Meckel dynasty and published literature on the history of their anatomical collections were analyzed and anatomical preparations from both of them were evaluated. To examine works and specimens of Ruysch and the Meckels, we implemented the historical-critical method, which involves the stages of acquisition of primary sources and literature, the critical evaluation of information contained in primary sources and the presentation of historical data in historical context in terms of objectivity and significance [16]. As the collections contain a large number of preparations, we made a selection of those preparations that relate to fetal development.

We have selected those preparations of fetal development of Ruysch and Meckel that allow us to make a comparison according to the following characteristics: (1) the appearance of the preparation and the anatomical techniques used to create it; (2) specimens of fetuses with abnormalities of physiological development (teratological specimens), their purpose and explanation of congenital abnormalities; (3) preparations that allow us to trace physiological processes during embryonic or fetal development. For each group, we selected three to five specimens. Thus, 8 specimens of the Ruysch collection and 11 preparations of the Meckel collections were selected according to the following criteria. On the one hand, they show various preparation techniques: wet preparations and dry ones (skeletons, mummified and skin preparations). The number of preparations from the Meckel collection is larger because in two cases several preparations were made from a single individual. On the other hand, we selected both typical preparations for collections (preparations of normal development and preparations with decoration in the case of Ruysch, and classic examples of osteological teaching preparations for fetal development at the time of the Meckels) and rare preparations (teratological). We also chose the most well-preserved preparations with which the anatomical techniques can be well traced. We also focused on similar preparations in both collections (joined twins, fetuses with placenta) to see the difference in their creation techniques.

Ruysch’s collection of about 800 preparations includes 93 preparations of fetuses from two to nine months, as well as a child of two years. The overwhelming number of preparations are wet and only eight preparations are dry: three skeletons and five mummified specimens. Most of the preparations represent the fetuses of normal development. At least nine specimens can be classified as teratological. As for the Meckel collections, it contains around 207 fetus specimens and about 70 embryo specimens. The exact number is difficult to determine because sometimes there are several fetuses or embryos in one jar, which has only one inventory number. In total, 113 preparations out of this number are teratological and were created by anatomists of the Meckel dynasty.

As for the Meckel collections, we should note that we selected the preparations regardless of the anatomist who had created them. This is explained, on the one hand, by the fact that the Meckels did not make an inventory of their private collections. Thus, it is not always possible to trace the exact provenience of the majority of their specimens. On the other hand, the anatomists themselves regarded preparations as an integrated whole. Meckel the Younger, who was especially focused on embryology and teratology and created the vast majority of teratological preparations, often used specimens of his father and grandfather for his research. Considering that not all preparations have survived to today, we also took into account the description of typical preparations, so that our sample would be representative. We chose those preparations that we were able to contextualize within the research of the anatomists. Photographs of all preparations from the Ruysch collection are available for research on the website of Peter the Great Museum of Anthropology and Ethnography (Kunstkamera) and published with permission. Photos of all preparations from the Meckel collections are retrieved from a database available at the Institute of Anatomy and Cell Biology at Martin Luther University Halle-Wittenberg. Table II from Meckel’s “Descriptio monstrorum nonnullorum cum corollariis anatomico-physiologicis” [17] in Figure 4B is retrieved from the Thüringer Universitäts- und Landesbibliothek Jena (ThULB).

## 3. Ethical Considerations

Since Ruysch’s and Meckels’ specimens were obtained prior to the era of informed consent, this raises questions about their ethical use. The creation and presentation of these specimens, which were lawful according to the principles of the time, appear to be unlawfully obtained according to our current assessment. For example, Ruysch often bought corpses of fetuses and stillborn babies with abnormalities from their parents or made a contract with them under which parents were allowed to visit his anatomical collection for free [3]. Meckel the Elder received corpses due to the legal regulations whereby the funerals of the poor were paid from the state, but their corpses had to be sent first to Meckel’s Anatomical Department [6]. However, Ruysch’s and Meckels’ specimens are those in which the memory of the former fetuses and infants has already faded and they do not touch the present life of descendants of their parents. As a result of the age of these specimens, anatomical techniques used for their creation, specific content and design are unique and of great value for the history of medicine. According to the criteria of the Stuttgart Recommendations of the German Medical Association on the handling of human tissue preparations in collections, museums and public spaces from 2003, human tissue preparations which are of particular importance to a specialist audience or the general public, may generally be used for presentation and demonstration purposes [18]. The recommendations draw attention to the importance of the anonymity of the demonstrated specimens and an optimal state of their preservation. Neither Ruysch’s nor Meckels’ preparations contain any personal information about the parents from whom the anatomists obtained the corpses of the fetuses and children. In order to preserve the preparations in an optimal condition, museum/collection staff conduct the restoration and re-conservation of preparations whenever necessary. This applies especially to the Ruysch collection largely composed of wet preparations, which need a careful re-conservation [19]. The most recent restoration of Ruysch’s specimens, conducted in 2003, helped to restore a number of them as close as possible to their original state and maintain their preservation [5].

Researchers who deal with the issue of the use of historical collections of embryonic and fetal specimens based on ethical considerations also suggest that these have to be maintained and appropriately used for research and education if a demand is justified [20,21].

In view of the above, we consider it possible to use selected preparations from the Ruysch and Meckel collections for historical research purposes, which emphasize the uniqueness of the preparations and their importance for a deeper understanding of the history of medicine. It should be noted here that the creation of anatomical preparations such as those made by Ruysch and the anatomists of the Meckel dynasty is not a current practice anymore. The use of human tissues for anatomical research purposes or medical training is possible when a person donates his or her own body, as well as in the case of perinatal autopsy, where a consent of the bereaved parents is required. Since the time of Ruysch and Meckels, anatomy teaching aids have been modernized so that anatomical preparations are not a necessity nowadays. Digital platforms, including 3D images and displays and 3D printed models are at the disposal of students. However, anatomical preparations are still an important part of research and visual aids used in the teaching process.

## 4. Results

### 4.1. Ruysch’s Selected Anatomical Preparations

**Figure 1:**
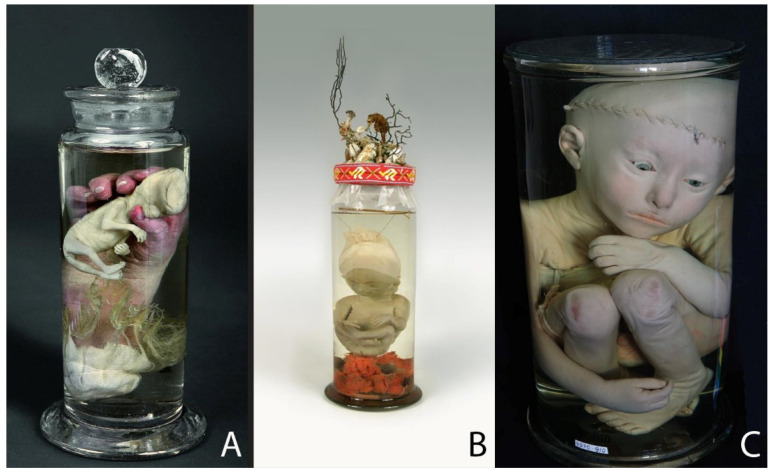
Specimens of normally developed fetuses and an infant: (**A**) Specimen 4070–784: an alcohol preparation with injection: a human fetus about 2 months old in the hand of a child of a few weeks old. The size of the jar: height—15.5 cm; diameter—6.8 cm. (**B**) Specimen 4070–799: 5-month-old fetus with a piece of injected placenta; a cap made of fabric with lace on the head. The size of the jar: height—27 cm; diameter—11 cm. (**C**) Specimen 4070–910: child about 2 years old with artificial eyes; the cranium and body cavities are opened. The size of the jar: height—42 cm; diameter—23.5 cm. © Peter the Great Museum of Anthropology and Ethnography (Kunstkamera), published with permission.

**Figure 2:**
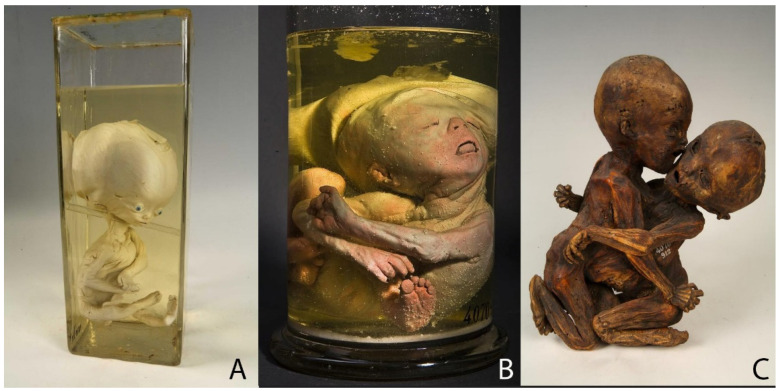
Teratological specimens: (**A**) Specimen 4070–917: spirit preparation: a male fetus of 8–9 months with congenital hydrocephalus. The size of the jar: height—42 cm; width—14 cm; depth—17 cm. (**B**) Specimen 4070–906: alcohol preparation with injection: a six-month-old fetus with intracranial fetiform teratoma. The size of the jar: height—26.3 cm; diameter—12.1 cm. (**C**) Specimen 4070–912: embalmed and mummified body of a conjoined twin. The size of the object: height—30.5 cm; width—16 cm; depth—9.5 cm. © Peter the Great Museum of Anthropology and Ethnography (Kunstkamera), published with permission.

**Figure 3:**
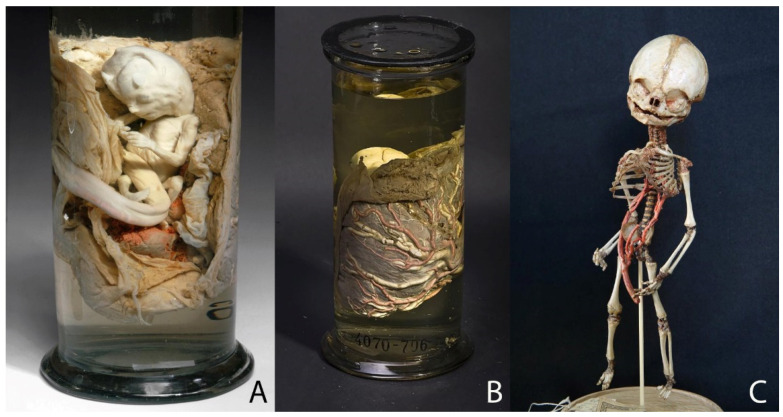
Specimens of fetal development: (**A**,**B**) Specimen 4070–706 (anterior and posterior view): 3-month-old human fetus with placenta injected with red (arteries) and white (veins) wax. The size of the jar: height—25 cm; diameter—12 cm. (**C**) Specimen 4070–864: skeleton of fetus about 5 months old on natural bands with umbilical cord and umbilical arteries. The size of the object: height—23 cm; width—5.5 cm; depth—4.9 cm. © Peter the Great Museum of Anthropology and Ethnography (Kunstkamera), published with permission.

We can divide Ruysch’s preparations of fetal development into those that show normally developed child fetuses (Figure 1), fetuses with physiological abnormalities (Figure 2) and those specimens in which Ruysch tried to show the physiological processes of fetal development (Figure 3). Most of those specimens are wet preparations, which were preserved in glass jars and an alcohol-based liquid containing 70% ethanol [19]. Many of the embalmed infants are decorated with lace or fabric (Figure 1A,B), or have inserted glass eyes (Figure 1A and Figure 3A). Ruysch also attached importance to the appearance of the entire preparation. As seen in Figure 1B, a lid of the jar is tied with a red ribbon and decorated with the composition made of embalmed seahorse, shells and plant twigs. The decorations on the jars were also made by Ruysch. It should be noted here that his daughter Rachel assisted him in his work, in particular making collars, cuffs and caps for the decoration of fetal and infant preparations [4,5,22]. Ruysch accompanied some preparations with philosophical or pathetic annotations. For one of the preparations that has not remained, similar to specimen 4070–799 (Figure 1B), he left the following inscription: “Oh how lucky I am, while my bones are resting like this!” [23] (p. 586). As we can see, and as has been established by experts, traces of red wax injections are evident on the specimens 4070–784, 4070–91, 4070–906 and 4070–706 (Figure 1A,C, Figure 2B and Figure 3A,B). Over the centuries, the injected skin has changed its color and darkened due to unfavorable storage conditions. The preparation 4070–910 (Figure 1C) looks almost the same as in Ruysch’s time: its injected covers have the appearance of being alive. Two of the eight selected specimens are dry: an embalmed and mummified body of a conjoined twin (Figure 2C) and a fetus skeleton on natural bands with a bunch of vessels (Figure 3C).

Three of the eight selected specimens are teratological preparations, which shows the following malformations: congenital hydrocephalus (a pathological enlargement of the fluid spaces (cerebral ventricles) of the brain filled with cerebrospinal fluid) (Figure 2A), intracranial fetiform teratoma (a very rare form of tumor that contains a variety of tissues which have human-like features and forms during embryological development) [24,25,26] (Figure 2B) and conjoined twin (Figure 2C). Regarding physiological processes, Ruysch was particularly interested in exploring the fetal circulatory system and thus did some preparations which show how the mother’s blood transfers across the placenta to the fetus (Figure 3A,B) as well as the circulation inside the fetus (Figure 3C).

### 4.2. Meckels’ Selected Anatomical Preparations

**Figure 4:**
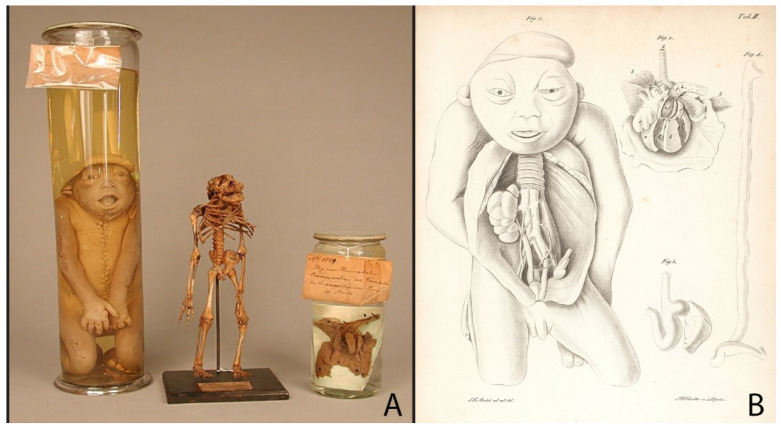
Specimens made by Meckel the Younger: (**A**) Specimens 114/2/2 (the size of the jar: height—39 cm; diameter—10 cm), 86/2/5 (the size of the object: height—22 cm; dimensions of the base plate—9.5 × 12.5 cm) and 119/2/2 (the size of the jar: height—17 cm; diameter—7.5 cm): skin preparation, skeleton and heart of a male fetus with neural tube defects: cranial malformation, spina bifida, hypertrichosis lanuginosa and left ventricular hypoplasia syndrome. Historical labels: “Nr. 1246. Hirnlose Missgeburt”; “N. 1592. Hemicephalus. Münter ppt.”; “Nr. 1509 / Herz eines Hemicephalen / Communication der Kammern / die Lungenschlagadern Zweige der Aorta. /105.” © Institute of Anatomy and Cell Biology at Martin Luther University Halle-Wittenberg, published with permission. (**B**) Table II from Meckel’s “Descriptio monstrorum nonnullorum cum corollariis anatomico-physiologicis”, 1826. © Thüringer Universitäts- und Landesbibliothek Jena, published with permission.

**Figure 5:**
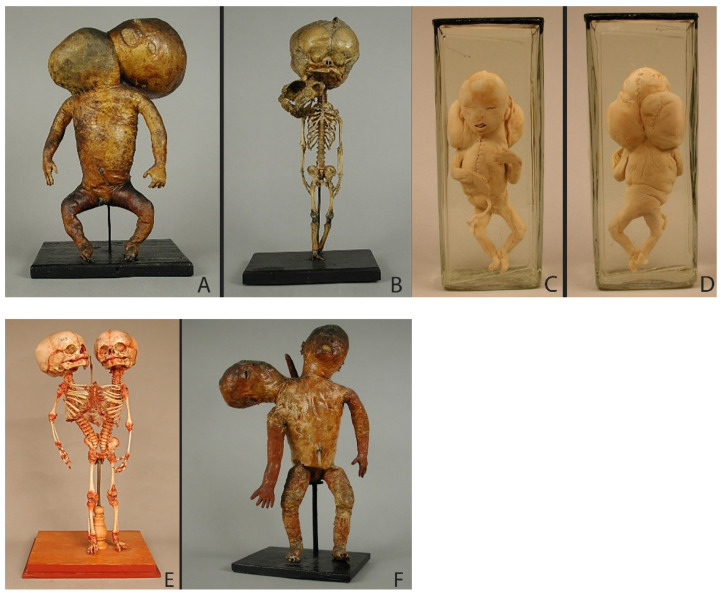
Teratological specimens: (**A**,**B**) Specimen 84/2/6 (the size of the object: height—36 cm; dimensions of the base plate—16 × 22 cm) and 84/2/7 (size of the object: height—39 cm; dimensions of the base plate—11 × 20 cm): skin preparation and skeleton of a male fetus with hydrocephalus and basal encephalocele (collection of Philipp Meckel) (**C**,**D**) Specimen 117/4/1 (anterior and posterior view): fetus with nuchal cystic hygroma (Ullrich–Turner phenotype), diagnosed by Meckel the Younger in his “Descriptio monstrorum nonnullorum cum corollariis anatomico-physiologicis”(1826) as “foetus tumoribus nuchae.” The size of the jar: height—24.5 cm; diameter—10 cm. (**E**,**F**) Specimen 83/1/4 (the size of the object: height—41.5 cm) and 85/1/1 (the size of the object: height—58 cm): skeleton and skin preparation from female dicephalic twins (tribrachius) (collection of Meckel the Elder). © Institute of Anatomy and Cell Biology at Martin Luther University Halle-Wittenberg, published with permission.

**Figure 6:**
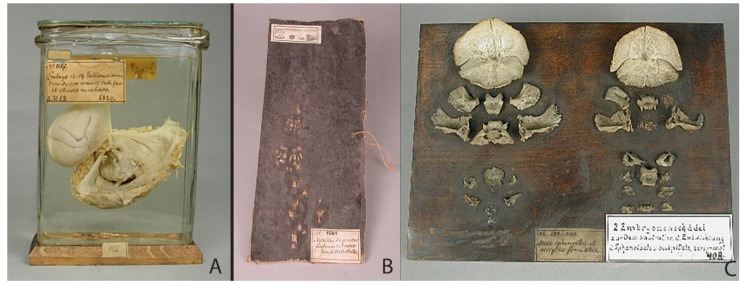
Specimens of fetal development: (**A**) Specimen 73/2/5: 12-to-14-week fetus with amniotic sac and placenta, preparation from the collection of Meckel the Younger, 1824. The fetus is completely enclosed by the amniotic sac and floats in its amniotic fluid. A glass ball keeps the preparation in suspension. Historical label: “No. 1157 / Embryo 13–14 hebdomadum cum sacco amnii integro et chorio morboso / 1824”. The size of the jar: height—19 cm; width—12.5 cm; depth—5.5 cm. (**B**) Specimen 47/5/3: upper jaws of fetuses, preparation from the collection of Meckel the Younger, 1820. Historical label: “Maxillae superiono foetuum 3–5 mens: Jan. 6. 1820. Meckel. The size of the plate: height—27 cm; width—14.5 cm. (**C**) Specimen 47/4/2: two so called “exploded” skulls of fetuses to demonstrate the development of the sphenoid and occipital bones. The exact year is not established. Historical label: “No. 289–290. / Ossis sphenoidei et.occipitis formatio. / 12.” The size of the plate: height—18 cm; width—23.5 cm. © Institute of Anatomy and Cell Biology at Martin Luther University Halle-Wittenberg, published with permission.

The fetal development preparations of Meckel the Younger, and also the preparations of his predecessors that he used in his research, can be divided into two categories: teratological preparations (Figure 4 and Figure 5) and specimens of physiological processes of fetal development (Figure 6).

The fact that Meckel the Younger created preparations for scientific and educational purposes can be seen in the example of the specimens 114/2/2, 86/2/5 and 119/2/2 (Figure 4A,B). From a single male fetus with neutral tube defects, he created three specimens: skin preparation, skeleton and heart. Supplemented with an engraving (Figure 4B), the preparations give a clear idea not only of the appearance, but also of the internal structure of the skeleton and organs of the fetus [27]. As other teratological preparations show, the Meckels used different techniques to create specimens (Figure 5). While a fetus with nuchal cystic hygroma was presented as a wet preparation (Figure 5C,D), fetuses with disorders which affect their bone developments were used both for skin and skeleton specimens (Figure 4 and Figure 5A,B,E,F). The wet preparations technique was used not only to capture the appearance of a fetus with developmental disabilities, but also to show fetal development and circulation (Figure 6A). In the collection of Meckel the Younger we can also see dry specimens of bones: upper jaws of fetuses (Figure 6B) and two so called “exploded” skulls of fetuses to demonstrate the development of the sphenoid and occipital bones (Figure 6C). No decorations of specimens can be found. Historical labels made in the post-Meckel era contain only the essentials: the number of the preparation and its brief description or diagnosis.

## 5. Discussion

### 5.1. Appearance of Specimens and Anatomical Techniques

#### 5.1.1. Ruysch’s “Elegant Anatomy”

Ruysch paid great attention to the aesthetic appearance of his anatomical preparations, as seen in the specimens 4070–784, 4070–799 and 4070–910 (Figure 1A–C). He covered the dissected and embalmed organs of the human body with lace of fabric, often embroidered with pearls, or put glass eyes into the orbitae of the embalmed infants. As he himself explained, it served two purposes: to cover the wound caused by amputation and to embellish the subject itself, “so that it would not be unpleasant to the eye, nor would it evoke neither fear nor disgust” [28]. It should be explained here that the 18th century was a time of the so-called “elegant anatomy”, when the notions of perfection, elegance, beauty and sensory perception were essential in the creation of preparations [29]. The regular use of the bobbin lace in Ruysch’s preparations can also be explained by its wide popularity in Europe as an important part of both men’s and women’s clothing of the time [29]. With the disappearance of lace from fashion in the late 18th century, anatomical objects with lace became an exception. Ruysch created many preparations in which children’s hands hold out body parts, organs, plants or fetuses. According to his explanation, in such specimens, a child’s hand either shows a skin structure or performs the function of presenting another bodily object, in our case, the human fetus [29]. Indeed, on one side, we see that the anatomist wanted to show the fetus, which is the center of attention, as if the hand was showing it to us. On the other side, it is the hand itself that attracts attention: it looks like it is still alive while the fetus became ghostly white. Ruysch could save the “healthy” glow of the hand due to wax injection containing a red pigment. The fetus without injection was influenced by the preservation liquid that caused its whitening. The mastery of the injection technique is especially evident in preparation 4070–910 (Figure 1C). The child, about 2 years old, with inserted artificial eyes still looks life-like and pinkish. Ruysch had to open the cranium and body cavities to be able to inject a mixture, which contained cinnabar, a natural source for the red pigment. It had long been believed that the mixture, carefully kept secret, allowed even the smallest blood vessels to be visualized, and thus gave the preparations of infants a lively expression, as if they were only asleep [5]. Another Dutch anatomist, Ruysch’s contemporary Jan Swammerdam (1637–1680), invented that injection method in the 1660s [29]. It was not the mixture that underlay the uniqueness of Ruysch’s preparations, but his skills. While injecting a preparation with the colored wax mixture, the latter has to remain warm and fluid, otherwise it congeals before reaching the smallest blood vessels. Adding some oil of turpentine can keep the wax liquid for a longer amount of time, but if too much oil is added, the wax will no longer be able to solidify at room temperature. Thus, this injection method is extremely difficult and requires the knowledge of the exact mixture of ingredients, right temperature, a lot of practice and meticulousness [29]. It should also be noted that the wet method of specimen preservation was unusual at that time due to the enormous cost of alcohol and glass jars [4].

#### 5.1.2. Meckel’s “Precise Anatomy”

Often the anatomists of the Meckel dynasty created several preparations from one individual in order to identify associated malformations. Unlike Ruysch, it was not about the aesthetic representation of the specimen, but about the precise examination of the individual in order to identify any additional malformed organs and thus also to collect teaching material for pathological-anatomical lessons. As an example, the fetus shown in Figure 4 originates from the collection of Meckel the Younger, who described the three preparations in his work *Descriptio monstrorum nonnullorum cum corollariis anatomico-physiologicis* in 1826 [17]. The wet skin preparation shows the condition sewn up again after the dissection, and only the heart of the fetus is preserved, so that the engraving made after Meckel’s drawings (Figure 4B) provides an additional insight into the internal structures of the fetus after dissection. It shows the fetus with the thorax open as well as its heart, spleen and intestine. The skeleton specimens are so-called natural skeletons in which careful maceration during preparation ensured that the ligamentous apparatus between the bones was preserved. Wet preparations were preserved with ethyl alcohol or turpentine oil [6].

The reason for the transition in the creation of “elegant” to “exact” specimens were changes in the development of anatomy. The publication and distribution of detailed handbooks on dissecting techniques has contributed to the dissemination of knowledge. Over time, the creation of anatomical preparations became more common. While at the beginning of the 18th century pathology was more of a theoretical discipline, by the end of the century it had become experimental as a part of anatomy teaching [24]. Therefore, the creation of preparations, although it continued to be part of the teaching and scientific process, was of lesser importance as proof of the anatomist’s exceptional skills and quest for elegance and perfection.

### 5.2. Teratological Preparations

#### 5.2.1. Ruysch’s Attempts to Explain Malformations

Since the concept behind the exhibition of Ruysch’s anatomical collection was that it had to be “pleasing to the eye” of visitors, preparations of deformities or “monstrous” babies were hidden in his cabinet and shown to the viewer only on request [4]. Although even creating a preparation of a neonate with malformation, Ruysch was guided by ideas of “elegant anatomy”: in the specimen 4070–917 (Figure 2A) he inserted both artificial eyes and a stick under the head of a child, so it did not sink to the bottom of the jar and remain visible from all sides. Infants and animals with malformations had long been a rare and important part of anatomical collections or so-called “cabinets of curiosities” in the 16th and 17th centuries. “Monsters” had been displayed as unique rarities, examples of “nature’s capriciousness” or God’s punishment for the parents’ sins [4,29]. It was also believed that a mother’s emotions and her mental state can influence the development of her child during the pregnancy. Ruysch was skeptical of that view and paid more attention to dysfunctions during the embryonal or fetal development as the primary cause of deformities [4]. Indeed, an opened cranial cavity reveals Ruysch’s attempt to understand the etiology of hydrocephalus. He is considered to be the first pathologist who came close to understanding the connection between hydrocephalus and spina bifida [30].

Ruysch diagnosed another specimen 4070–906 (Figure 2B) with hydrocephalus, too. As the delivery of the baby proved difficult, the midwife called Ruysh to help. By the time he arrived, she had already succeeded in removing the fetus and placenta. To the astonishment of Ruysch, the midwife pulled out of the uterine cavity an agglomeration of miniature extremities, such as arms or legs. Those unconnected body parts appeared to belong to a child of around 3 months of gestation. The baby was most probably stillborn. Ruysch concluded that at the moment of conception, several ova had been impregnated and entangled with each other while moving into the uterine cavity [23]. The phenomenon Ruysch described is a rare congenital disorder called intracranial fetiform teratoma—a tumor which forms during embryonic development [24,31]. Although Ruysch did not explain the opening in the child’s head, the researchers suggested that tumorous tissue came out of the skull ruptured during delivery, as has already been reported on several cases of fetiform teratoma [25,26,31].

The teratological part of Ruysch’s collection contained not only wet but also dry preparations and skeletons, mostly conjoined twins. Figure 2C shows prematurely born children joined together at the thorax and abdomen. He received the dead children from their parents in exchange for letting them and their friends come see the twins as often as they wanted [4,31]. Ruysch did not explain why he preferred to embalm and mummify the twin instead of making a wet preparation.

#### 5.2.2. Investigation of Malformations by Meckel the Younger

Teratological preparations also occupied an important place in the Meckel collections. One of the greatest achievements of Meckel the Younger was in the field of pathology. His *Handbuch der pathologischen Anatomie* (Manual of Pathological Anatomy) [32] became a standard work of teratology in the 19th century which was translated in French and English [33]. The aim of his book was to study the deviations from norm, the malformations, which he divided into two categories: deviations of form and deviations of texture. While the first may arise in the early period of embryonic and fetal development, according to Meckel, the second occurs mainly in later periods of life. His main research focus was deviations of the form. The very significant deviations from the usual form he defined with the term “monstrosity”, and the minor deviations in formation with the terms “natural games” or “varieties”. For example, regarding hydrocephalus, Meckel the Younger speaks of an inhibition formation (“Hemmungsbildung”), the development of which he tries to explain with an inhibition as a form of a phylogenetic preliminary stage [27,32]. He noted that most primary malformations are anomalies of incomplete development at an embryonic or fetal stage of development [34]. To describe a hydrocephalus, Meckel the Younger went back to his father’s preparations 84/2/6 and 84/2/7 (Figure 5A,B) and described the skull in his *Handbuch der pathologischen Anatomie* [32]. The same specimens of the fetus which we now diagnose with basal meningoencephalocele [27] served as research objects for the student of Philipp Meckel, Friedrich Gotthilf Voigtel (1769–1813) [35].

The preparations from the Meckel collections are still used today for teaching and research. For example, specimen 117/4/1 (Figure 5C,D) was re-examined from a pathomorphological point of view and was diagnosed as Ullrich–Turner “phenotype” [34,36]. The fetus was described by Meckel the Younger as “foetus tumoribus nuchae” [17], by which is currently meant a cystic hygroma or benign tumor that appears as a fluid-filled sac often forming in a newborn’s head or neck area. All in all, three fetuses that are preserved in the collection are definitively described by Meckel the Younger in 1826 and diagnosed as “foetus tumoribus nuchae”. Considering the results of our investigations, observations on fetal nuchal cystic hygromas can be traced back to Samuel Thomas Sommerring’s work *Abbildungen und Beschreibungen einiger Misgeburten, die sich ehemals auf dem anatomischen Theater zu Cassel befand* (Illustrations and descriptions of some miscarriages, which formerly belonged to the Anatomical Theatre in Kassel) [37]. Meckel also mentions Adolph Wilhelm Otto (1786–1845) reporting such cases in 1824 [17]. Of the fetuses with nuchal hygroma colli or the Ullrich–Turner phenotype that are historically documented in the literature, only those from the Meckel collections have been preserved [6,36].

### 5.3. Fetal Development

#### 5.3.1. Ruysch Research on Embryology and the Fetal Circulatory System

As an obstetrician, Ruysch created many preparations of embyos and fetuses in all stages of growth in order to show the development of a human being. His collection included a range of preparations, from embryos of the size of a grain of sand to full-term babies [4]. Ruysch was one of the first anatomists who demonstrated successive stages of embryonal and fetal development [23,38]. Through the visualization of embryos as anatomical preparations, he was able to contradict the opinion of some of his colleagues, who believed that “they could see in a woman’s egg a small creature with arms, legs and a head” [22]. However, Ruysch could show that the embryo in the very early stage of its development was merely a “formless white lump” [23].

His specimens of embryos in the first weeks of pregnancy that impressed his contemporaries so much have not remained to the present day [22]. Ruysch’s preparations have not escaped the common fate of many anatomical preparations. Due to unfavorable storage conditions, such as evaporation or even complete leakage of liquid, decreased strength of alcohol, dampness and moths in dry preparations, specimens inevitably deteriorated and were excluded during inspections. The earliest fetuses that have remained in the Ruysch collection are two to three months old.

Ruysch also studied fetal circulatory system. Although blood circulation was discovered in 1628 by the English physician William Harvey (1578–1657), there was no exact knowledge about fetal blood circulation. Ruysch’s alcohol preparation of a 3-month-old fetus with placenta injected with red (arteries) and white (veins) wax (Figure 3A,B) is an example of one of the few specimens with injected placentas, due to which the blood vessels are clearly visible. Ruysch injected umbilical arteries with red pigmented wax and the umbilical vein with white wax to show the circulatory system of a fetus. He correctly considered it to be essential for the healthy development of a child [38]. Because of his careful technique of injecting the placenta, he found that it consisted of vessels, not glands, as he had previously assumed [23,38]. Ruysch also created dry specimens which presented the circulation inside the fetus.

#### 5.3.2. Fetal Bone Studies of Meckel the Younger

Turning to the preparations of fetal development created by Meckel the Younger, we see that some of them were created for educational purposes, because they did not carry any new knowledge of the time. These include preparations of fetuses in the maternal placenta (Figure 6A). This preparation is certainly skillfully performed and represents a rare opportunity to show the fetus completely enclosed by the amniotic sac and its connection to the maternal placenta. On the other hand, Meckel’s preparations were also devoted to a direction in fetal development research at that time, namely the fetal bone study: the upper jaws of fetuses, and two so called “exploded” skulls of fetuses to demonstrate the development of the sphenoid and occipital bones. These preparations were used to follow the formation, development and maturation of the skeletal system as well as to illustrate the bone structure of fetuses and their pathologies [6]. In 1820, Meckel the Younger discovered a primary lower jaw, which remained only in embryos, called *Meckel’s cartilage* after him. With the development of the bone skeleton, Meckel’s cartilage is moved away by the mandibular bones that develop around it, and is reduced, giving origin to some auditory ossicles. Through this and other research, Meckel refuted the common view of the preformationism, according to which an organism develops from a fully formed miniature version of itself that has existed since the beginning of creation [6].

## 6. Conclusions

As a result of comparing the Ruysch and the Meckel collections, we can draw several conclusions. Regarding the appearance of the preparations and the anatomical techniques used to create them, we should note a transition from the “elegant” preparations (Ruysch) to exact ones (Meckel). While in his time, Ruysch’s preparations were compared to works of art, Meckels’ specimens were primarily created for teaching and research purposes. However, Ruysch’s collection was not simply a collection of skillfully created preparations that were equated with objects of art or a “cabinet of curiosity”. As we see in the preparations tracing the fetal circulation, they served research purposes and were scientific discoveries of the time. As for Meckels’ preparations of fetal development, we see both preparations of physiological processes already known at that time and experimental ones, which in turn confirms the educational and scientific purposes of their creation. Regarding teratology, both Ruysch and Meckel the Younger tried to explain malformations, but their anatomical preparations could hardly provide answers to the cause of deviations from norm. Nevertheless, they provided descriptions of the malformation and thereby accumulated new knowledge.

The differences between the collections can be explained not only by the different stages of development of anatomy and medicine of the time, but also by the research interests of the anatomists themselves. Unlike the Meckels, comparative fetal osteology was not a subject of Ruysch’s research, so we do not find such osteological preparations in his collection. Moreover, fetal blood circulation, sufficiently explored by the 19th century, was not a research focus for anatomists of the Meckel dynasty. The numerous teratological specimens in the Meckel collections can also be explained by the fact that at that time pathology was developing as an independent discipline. Thus, teratological specimens were an essential teaching aid for medical students. The goals of the collection and the scientific interests of the anatomists, in turn, determined the composition and choice of anatomical techniques in the creation of the specimens.

## Data Availability

Not applicable.
